# Phasetime: Deep Learning Approach to Detect Nuclei in Time Lapse Phase Images

**DOI:** 10.3390/jcm8081159

**Published:** 2019-08-02

**Authors:** Pengyu Yuan, Ali Rezvan, Xiaoyang Li, Navin Varadarajan, Hien Van Nguyen

**Affiliations:** 1 Department of Electrical and Computer Engineering, Cullen College of Engineering, University of Houston, Houston, TX 77004, USA; 2 Department of Chemical and Biomolecular Engineering, Cullen College of Engineering, University of Houston, Houston, TX 77004, USA

**Keywords:** T-cell, nuclei, phase image, fluorescent imaging, instance segmentation

## Abstract

Time lapse microscopy is essential for quantifying the dynamics of cells, subcellular organelles and biomolecules. Biologists use different fluorescent tags to label and track the subcellular structures and biomolecules within cells. However, not all of them are compatible with time lapse imaging, and the labeling itself can perturb the cells in undesirable ways. We hypothesized that phase image has the requisite information to identify and track nuclei within cells. By utilizing both traditional blob detection to generate binary mask labels from the stained channel images and the deep learning Mask RCNN model to train a detection and segmentation model, we managed to segment nuclei based only on phase images. The detection average precision is 0.82 when the IoU threshold is to be set 0.5. And the mean IoU for masks generated from phase images and ground truth masks from experts is 0.735. Without any ground truth mask labels during the training time, this is good enough to prove our hypothesis. This result enables the ability to detect nuclei without the need for exogenous labeling.

## 1. Introduction

The dynamic observation and measurement of molecules, subcellular organelles, cells and collections of cells is best accomplished using microscopy. The direct observation of subcellular structures and organelles within cells is challenging. Fluorescently labeled antibodies, fluorescent proteins, and small molecules have become indispensable tools for the visualization of cellular organelles with high contrast and resolution, leading to major advances in our understanding of how cells and organelles function.

The use of these fluorescent molecules, however, has its own set of challenges. Fluorescent antibodies cannot be used with live cells for examining subcellular features; the use of the fluorescent proteins requires genetic modification of the cells and appropriate proteins, and the specificity and reproducibility of small molecule fluorescent labels can be non-uniform across cells and cell types. Furthermore, with respect to time-lapse imaging microscopy, fluorescent labels are prone to photobleaching and can generate toxic byproducts of degradation upon repeated exposure to light. Studying multiple subcellular features requires labeling with multiple dyes which in addition to multiplying the problems listed above, also necessitates an elaborate optical set up that can reliably distinguish and differentiate the different fluorescent labels used in the experiment. Lastly, although most optical setups and downstream image processing algorithms have been optimized for the study of adherent cells, the study of lymphocytes engenders its own challenges since the cells are non-adherent and are capable of inherent locomotion at speeds varying from 4–10 microns/minute [1]. The ability to sample large numbers of lymphocytes requires the need for high-throughput imaging with fast acquisition rates [2,3]. This presents yet another set of challenges since individual images are noisy, might not be out of focus (movement of lymphocytes), unevenly illuminated and contain other artifacts that arise during high-throughput time lapse imaging [2,4].

We hypothesized that the phase contrast microscopy images contain sufficient information to be able to identify and track nuclei as an example of subcellular organelles within lymphocytes. Although there have been examples of machine learning being utilized to identify subcellular organelles within live cells [5], these have been restricted to adherent and non-motile cells. The contributions of our work are summarized as:

1. Establish a workflow combining traditional blob detection method and modern deep learning method to do nuclei instance segmentation which does not require labeled masks during the training time.
2. Detect and segment the nuclei based on the phase contrast microscopy images instead of stained channel images.
3. Perform the dynamic T-cell fluorescent imaging experiments, train a detection/segmentation model on the real data and quantify the validity of the results.

## 2. Materials and Methods

### 2.1. Immune Cell Data Generation

#### 2.1.1. CAR T Cell Manufacturing

T cells genetically modified to express a CD19-specific chimeric antigen receptor (CAR T cells) were generated from the peripheral blood mononuclear cells (PBMCs) of a healthy volunteer donor obtained via Gulf Coast Regional Blood Bank (Houston, Texas) following approved protocols by the Institutional Review Boards at the University of Houston and the University of Texas MD Anderson Cancer Center. Second-generation CD19-specific CAR T cells (designated CD19/4-1BB) were maintained in complete culture medium RPMI (Corning, Corning, NY, USA) + 10% fetal bovine serum (FBS) (ATLANTA biologicals, Hall County, GA, USA) (R10) along with cytokines (IL2 (Prometheus, San Diego, CA, USA) and IL21 (Peprotech, Rocky Hill, NJ, USA)). The cells were phenotyped and tested in functional assays routinely, as described previously (Liadi et al., 2015) [2].

#### 2.1.2. Fluorescent Staining of Nucleus

CAR T cells were incubated at 37 °C for 30 min in 1:1 (*v*/*v*) solution of live cell staining buffer (Abcam, Cambridge, UK) and RPMI (Corning, Corning, NY, USA) containing final concentration of 1 μM Hoechst 33342 (Sigma, St. Louis, MO, USA) for labeling nucleus. Cells then were washed with Hanks’ balanced salt solution (Cellgro, Mediatech Inc., Manassas, VA, USA) + 10% HEPES (Corning, Corning, NY, USA) first and complete culture medium RPMI + 10% FBS (R10) for the second wash.

#### 2.1.3. Nanowell Array Fabrication

Master template of nanowell array was designed using AutoCAD (2012, Autodesk, San Rafael, CA, USA) as described previously [2,3,6] and fabricated on a silicon wafer using soft lithography techniques. Nanowell array was fabricated by spinning silicon wafer poured with a Polydimethylphenylsiloxane (PDMS) mixture at 1000 rpm for 30 s and then baking at 80 °C for 3 h in an oven. The dimension of each nanowell was 50 μm × 50 μm. After detaching nanowell array from the master, it was air plasma oxidized and attached to the bottom of a 50-mm glass bottom petri dish. Nanowell array was plasma re-oxidized prior to use.

#### 2.1.4. Timelapse Imaging Microscopy in Nanowell Grids (TIMING)

**Figure 1:**
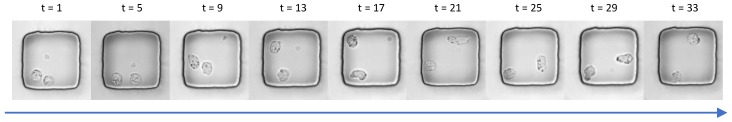
Representative phase contrast images of T lymphocytes within a nanowell. Each microscopy image contains 36 nanowells and is acquired at 35 timepoints (*t*).

TIMING assays were performed essentially as described previously [7]. Briefly, CAR T cells were loaded on a nanowell array fixed on a 50-mm glass bottom petri dish. 1.5 mL R10 media was added to the petri dish after loading the cells on the nanowell array. An inverted fluorescent microscope (Zeiss Axio Observer Z1; Zeiss, Oberkochen, Germany) equipped with a motorized stage, Lambda-DG4 illumination system, differential interference contrast (DIC) condenser annulus, a 20× Zeiss Plan-Apochromat, air, 0.8 NA objective and a scientific CMOS camera (Orca Flash 4.0, Hamamatsu, Shizuoka, Japan) was used for imaging. DAPI was used for detection of nuclei. Images were acquired for 3 h with 5 min intervals. Time lapse images are shown in Figure 1.

#### 2.1.5. Ground Truth

The whole dataset contains 120 blocks and there are 36 nanowells for each block. For nuclei instance segmentation task, we used 60% of blocks as our training data and the rest as our test set. As our mask generated by the blob detection is not the ground truth, we asked the experts biological field to annotate nuclei masks of 200 cells from the test dataset. **This data set is called golden mask dataset**. It can be used to evaluate both blob detection algorithm and the nuclei segmentation branch in the Mask RCNN method.

### 2.2. Image Data Preprocessing and Network Configuration

**Figure 2:**
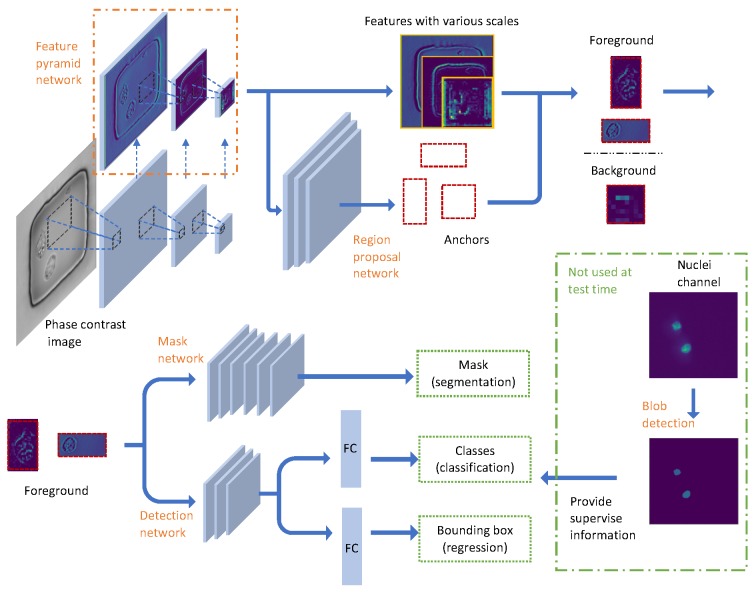
The workflow of instance nuclei detection and segmentation. There are five modules: Blob detection, Feature pyramid network, Region proposal network, Detection network and Mask network. Note that blob detection is only utilized for training and is not needed during testing.

In this work, we only tried nuclei as an example of subcellular organelles. Our workflow as shown in Figure 2 has two main steps: generate binary masks from nuclei stained images using traditional unsupervised blob detection method; use these masks to train a Mask Regional Convolutional Neural Network (Mask RCNN) [8] to do nuclei detection and segmentation based on phase images. The first step is considered as the data preprocessing/preparation for the second step. The Mask RCNN method then can be splitted up to 4 parts: scan the images and extract feature with Feature pyramid network; find interested foreground region at different scales; detect and classify the object; generate instance masks for each object. To be noticed, Mask RCNN is pretrained on MS COCO [9] dataset and after the Mask RCNN network is trained, no nuclei channel image is needed during the test time. The implementation of Mask RCNN used [10] as a reference and both two steps used python as the programming language.

#### 2.2.1. Binary Mask Generation

To generate the masks for training, we implemented cell segmentation on grayscale fluorescent images based on a general pipeline of blob detection in scikit-image toolbox [11]. First, min-max normalization is performed for all the fluorescent images to map the intensity of the images to the range 0 to 1. After that, Otsu’s thresholding method [12] is used to separate the foreground and background pixels, such that the foreground may consist of isolate components (only one cell in each component) and separated connected components (more than one cell in each component). Keep in mind this step is also called semantic segmentation [13] in general deep learning problems, which is only able to tell the existence of the cell while unable to differentiate one cell from another if they are too close to each other. Then for each connected components, we used Laplacian of Gaussian (LoG) [14] to detect the centroid of the cells and watershed segmentation [15] to outline the exact border of each cell. Finally, the mask of the fluorescent image is the union of isolate cells and the separated connected cells.

Note that the plain blob detection method tends to generate larger masks compared to the exact border of the cells. So we implemented several pixels of erosion to shrink the instance masks from the pipeline above. The experiments show that the eroded mask trained the network better in terms of pixel-based performance. The comparison of the erosion degree for binary mask generation can be found in the results Section 3.1.

Generating masks from stained channels is relatively simple compared with from the phase contrast images as we can observe the clear boundary for each object. But detecting nuclei from the phase contrast images is much more difficult. Because the different organelles are displayed together, the boundary of the nuclei can be very ambiguous. And because the cells are moving or dying, their body shapes vary from cell to cell. Mask generated by blob detection method can be served as the supervised signals for training the following detection and segmentation model.

#### 2.2.2. Pyramid Feature Extraction

From here starts the Mask RCNN structure. The standard Resnet50 networks [16] are used as the feature extractor. The resnet structure has special resnet blocks which make the network more efficient by learning the residual of input and output. More importantly, it can learn identity function by simply setting residual to be zero, making it easier to converge. Resnet has a total of 50 layers and 5 stages (we only draw 3 stages in Figure 2 for simple illustration). The size of images in the latter stage is half of the previous stage while the number of the feature layers will be double. Same as the fluorescent images, the phase contrast images are normalized with min-max normalization. It is useful to reduce the influence of intensity fluctuations of the light source on the detection results. The input microscope phase image has a size of 2048 × 2048 × 1, the size of the nanowells is around 250 × 250 and the size of cells has a range from 20 to 60 pixels. As the cells are so small in the whole images and the cells are also very sparse, we upsampled the input images by a factor of 2. It is achieved with Bi-linear interpolation implemented in scikit-image toolbox [11]. After that we cropped patches of 512 × 512 (similar size as the nanowells) from the 4096 × 4096 phase images to make the cells larger. The binary masks are processed in the same way before sending to the networks. With the root feature number of 64, the feature maps in the highest stage have a size of 16 × 16 × 2048. Different stages extract features at different scales. Feature pyramid network (FPN) is introduced by [17] to have a better representation of the input image by adding another pyramid which will pass the higher level features back to the lower levels. Thus the FPN has two paths of data flow: bottom to up and up to bottom. As we go up, the spatial resolution decreases, but for each node in the feature maps, it represents the object in a larger area. As we go down, the semantic meaning of the lower level features is enriched by indirectly communication between pixels in a larger area. There are skip connections between two paths to make the training easier. Those five-stage feature maps are used as the input for the following Region proposal networks (RPN).

#### 2.2.3. Proposal Generation

The proposal can be seen as the foreground of the images. The purpose of this step is to distinguish between foreground (FG) and background (BG). In order to generate proposals, we need to scan the whole images. The regions that are scanned over are called anchors. Anchors are predefined and their sizes are dependent on the size of object we are going to detect. In the nuclei detection case, the anchors have five different size scales: $8^{2},16^{2},32^{2},64^{2},128^{2}$ pixels. Because the largest cell has a length of 120 pixels after factor-2 upsampling, these five size scales anchors can capture all the possible cells. The step size of scanning is half of the anchor size, so that anchors are overlapping even for the same size scale. And there are three aspect ratio {1:2, 1:1, 2:1} of anchors for each position to make sure both tall and wide shapes can be detected. The region proposal network will scan over the feature maps instead of the original images to reuse the extracted features efficiently and avoid duplicate calculations. The outputs of RPN are the probabilities of being FG and the adjustment of the anchor boxes. Both the center and the size of Anchor boxes can be changed a little bit after the adjustment. The anchors with high FG probabilities or contain an object are positive anchors while the anchors with high BG probabilities are called negative anchors. Both positive and negative anchors are used for training RPN. With the RPN predictions, we pick the top anchors that are likely to contain objects and refine their locations and sizes. If several anchors overlap too much, we keep the one with the highest foreground score and discard the rest (referred to as Non-max Suppression [18]). After that we have the final proposals (regions of interest) that we pass to the next stage.

#### 2.2.4. Nuclei Detection

Given final proposals and the nuclei bounding box from blob detection, we are able to train a classifier and a detector of nuclei. If there are more organelles included, we just need to train more classifiers here. Just like RPN, the classifier will predict the probabilities of the regions to be nuclei or the background. If the region is predicted as background, it will be discarded. Otherwise, the probability will be referred as prediction score as one of the output result. Because the final proposals have different size and different scale, the inputs of the classifier coming from a different pyramid feature layer have different sizes. An alignment layer is needed to keep the size of the input consistent (7 × 7 in the experiment, can be changed due to computational ability). The detection task is realized by matching the predicted bounding box to the blob detection bounding box of nuclei. Both classifier and regressor use 2 fully-connected (FC) layers before the last layer. Multi-class cross entropy loss is used for training classifier and smooth *ℓ*-1 loss is used for the bounding box regressor.

#### 2.2.5. Nuclei Segmentation

The final step is to add a mask network to produce the mask for each nucleus. This mask network is a fully convolutional network and will only take the positive regions selected by nuclei classifier. Same as the detection branch, the feature proposals as the input of mask network are resized to 14 × 14 after an alignment layer. The masks (with a size of 28 × 28) are represented by float numbers instead of binary numbers, making the boundary more accurate when scaled to a different size. The size of the input and output for the mask network can be modified. Larger size may give better results but also requires more computational resources. The mask branch is implemented with 4 convolutional layers with kernel size 3 × 3 and 256 feature maps for each layer. The binary cross entropy loss is used to train the mask network. The masks will be scaled up to original size and that’s what we want from only observing the phase images.

### 2.3. Metrics for Performance Evaluation

#### 2.3.1. Intersection over Union (IoU)

The Jaccard index, also referred to as the intersection-over-union score, is commonly used in the evaluation of semantic segmentation:

(1) $$J_{c}\left(\hat{y},y^{*}\right)=\frac{\left|\left\{\hat{y}=c\right\}\cap \left\{y^{*}=c\right\}\right|}{\left|\left\{\hat{y}=c\right\}\cup \left\{y^{*}=c\right\}\right|}$$

where *c* is the nucleus class, $y_{i}^{*}\in \left\{0,1\right\}$ is the label of pixel *i*. $y_{i}^{*}=1$ means pixel *i* is in nucleus area. $\hat{y_{i}}\in \left\{0,1\right\}$ is the prediction for pixel *i* of whether it belongs to class *c*. IoU is very useful when we want to evaluate the overlapping percentage of two objects. It is also used in the next subsection when we compute the average precision.

#### 2.3.2. Average Precision (AP)

For object detection problem, there are two tasks we need to do:

1. Determine whether an object exists in the image.
2. Find the corresponding location if the object exists.

The IoU can serve as a good indicator for segmentation, but it cannot tell us how good are our detection results. We use the *average precision* (AP) to measure the quality of our detector. Suppose there are two sets, set of labeled objects $S_{l}$ and predicted objects set $S_{p}$. Given an IoU threshold $T_{iou}$, if two objects from different sets match in the way that $IoU\left(x,y\right)>T_{iou},\;\mathrm{for}\;x\in S_{l}\;\mathrm{and}\;y\in S_{p}$, then this is considered as a True positive (TP) example. The samples in $S_{l}$ but not in $S_{p}$ are False Negative (FN) and the samples in $S_{p}$ but not in $S_{l}$ are false positive (FP). Precision and recall are computed by

(2) Precision=TP/(TP+FP),Recall=TP/(TP+FN)

And the precision-recall curve is obtained by adjusting the prediction score threshold $T_{s}$. All predicted object with prediction score lower than $T_{s}$ will be eliminated from the prediction set $S_{p}$. The average precision is the mean value of precision under different $recall_{j}$. For each IoU threshold, compute the mean of AP over *N* images will give us the final indicator for the detection:

(3) $$AP=\frac{1}{N}\sum_{i=1}^{N}\frac{1}{M}\sum_{j=1}^{M}Precision_{i}\left(Recalls_{j}\right)$$

Note that here $Precision_{i}$ is a function. In this way, this metrics is associated with a prediction score for each object and the model is evaluated as different level of confidence.

## 3. Results

### 3.1. Mask Label Generation Accuracy

**Figure 3:**
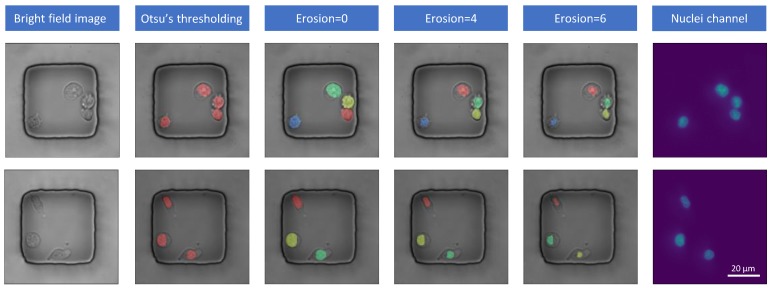
Comparison of representative masks generated from nuclei channel with blob detection methods.

From the DAPI stained nuclei channel, we can get the grayscale images. The first task is to generate accurate binary masks for nuclei based on these grayscale images. The blob detection method can control the size of the mask by a hyperparameter erosion degree. Using the golden mask dataset, we compared the accuracy of the generated mask by Intersection over Union with different erosion degree. For the blob detection method, we use erosion degree of 0, 4 and 6 (We use E4 as the abbreviation of blob detection method with an erosion degree as 4). Ostu binarization result is also added as the baseline. The mean IoU results are shown in the Table 1. As we can see from the results, when the erosion degree is 4, the generated masks have the largest overlapping with the ground truth masks. Refer to Figure 3, E0 and Otsu methods tend to generate bigger masks than the ground truth while masks generated with E6 are too small. From this observation, we use E4 to generate masks for experiments in the rest of this paper.

### 3.2. Nuclei Detection Accuracy

This result is to show the nuclei detection accuracy by computing the IoU between the nuclei bounding boxes or masks generated from the phase contrast image and that generated from the stained nuclei channel. Because we do not have the ground truth masks based on the whole images, the blob detection results are used as the ground truth for evaluating detection accuracy. As observed in the previous section, masks from the blob detection have significantly high overlap with those in the golden set. Therefore, detection accuracy based on the masks generated by the blob detection is expected to strongly correlate with the accuracy based on the masks annotated by human experts.

**Figure 4:**
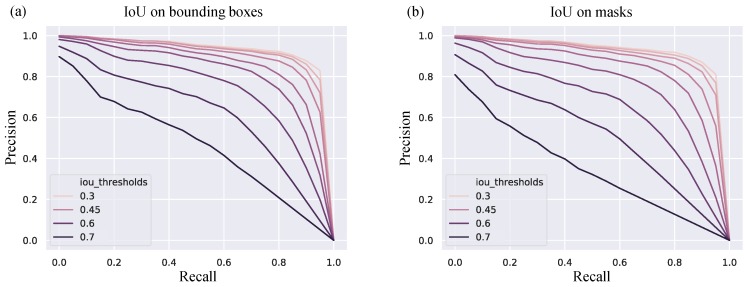
Precision-Recall curve with various IoU thresholds. (**a**) IoU is calculated based on bounding box of ragion of interests (**b**) IoU is calculated based on masks.

We set a threshold of prediction score by the Mask RCNN method to reduce the number of false positive. By varying this hyperparameter, we found that set the threshold as 0.9 can give us the best average precision score. The detection algorithm was running with IoU threshold from 0.3 to 0.7 with 0.05 interval and the results are shown in Table 2 and Figure 4. When the IoU requirement is 0.5, the algorithm can get the average precision of 0.821, which means 82.1% of predictions are truly nuclei. This detection results can be further improved if we have a higher quality phase contrast images.

**Figure 5:**
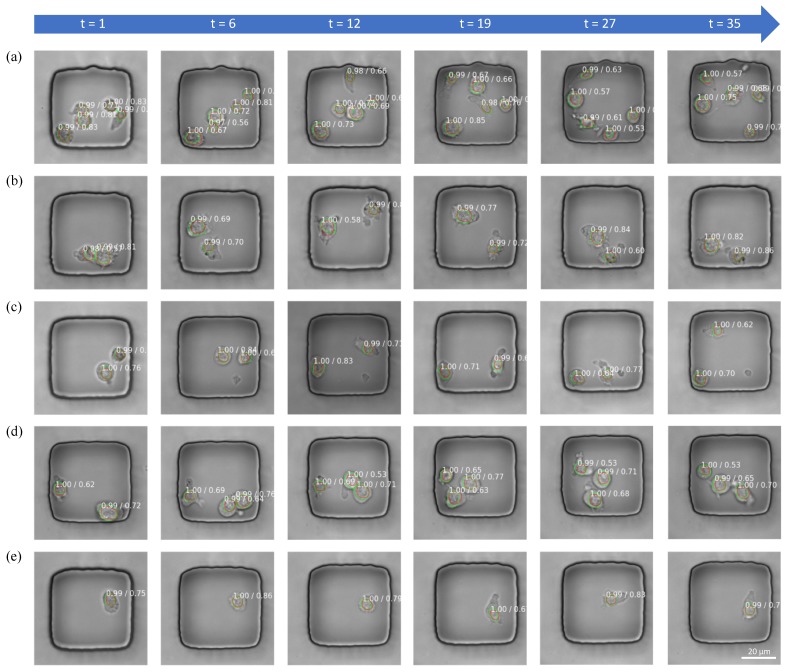
Representative examples of the results of nuclei detection within T cells in 5 nanowells; the annotated number is the prediction score/IoU over the mask. Green lines and red lines denote masks from blob detection and Mask RCNN, respectively. *t* is the time slot for each phase image being recorded. (**a**–**e**) are 5 different nanowells.

The detection sample results are shown in Figure 5. Green lines are the boundary of masks generated from nuclei channel using blob detection method. Red lines are the boundary of masks predicted by the Mask RCNN. The annotated number means prediction score/IoU over the mask.

For most detected nuclei, the prediction score given by the network is high. This means that the detector has high confidence about the presence of nucleus. From the samples we can see that the algorithm can detect the location and the shape of the nucleus consistently over time.

### 3.3. Nuclei Segmentation Accuracy

Previously average precision based on the masks can give us a clue of how accurate the masks generated from phase image are. However, the masks we used to calculate average precision is from blob detection method. As we can see from the mask generation accuracy results, they still have some error compared to the ground truth masks annotated by the experts (IoU 0.842). Thus, directly computing the average IoU will give us a more reliable evaluation result on the mask branch. The average IoU on 200 golden test set is **0.735**, and the standard deviation is 0.058. Given that the label we used for training Mask RCNN has a IoU of 0.842, and generating masks from phase contrast images is much more difficult than generating masks from nuclei channel, the current result is reasonably good. More discussion on the error and challenges can be found in the next section.

**Figure 6:**
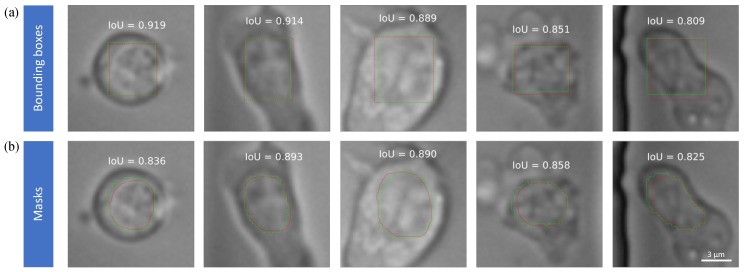
Representative examples of high accuracy (**a**) bounding boxes and (**b**) masks for single cells. Green lines and red lines denote bounding boxes/masks of the nuclei from experts and Mask RCNN, respectively. IoU as a detection metrics is annotated in each subfigure.

In Figure 6, we can see that good segmentation mask has an IoU of more than 0.8 compared with ground truth form golden test set. And in most situation, the IoU of the bounding boxes is even higher. The boundary of the ground truth is a little bit ruffled, the reason is that we do an interpolation of 2 to make each single cell bigger, and the mask prediction is running on the interpolated images.

### 3.4. Error Analysis

There are two steps for the Mask RCNN inference. The first step is to correctly detect nuclei in certain regions. The second step is to perform segmentation on that location. Thus we are going to discuss the error and challenges in these two parts.

**Figure 7:**
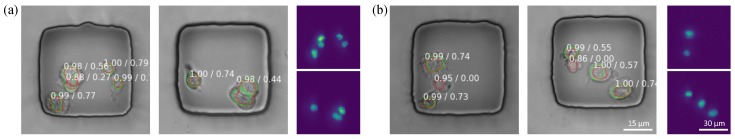
Representative examples of errors in detection. Green lines and red lines denote masks from blob detection and Mask RCNN, respectively. Blue images are nuclei channel from biological experience. (**a**) Mask RCNN wrongly merged two nuclei into one because of the mask label error; (**b**) Mask RCNN raised false positive prediction when the cell extends its body.

Figure 7 shows the blob detection masks (green lines), Mask RCNN predicted mask (red lines) and the nuclei channel from biological experience (blue images). From Figure 7a, we see that when two cells are very close to each other, the algorithm may make mistakes. The blob detection method incorrectly classifies two adjacent nuclei as a large nucleus for the image on the left. Because some of the training labels are incorrect, Mask RCNN wrongly merged two nuclei into one. If we can improve the accuracy of the masks label, the inference result can be improved. From Figure 7b, we see that the algorithm may raise false positive prediction when the cell extends its body, making it likely that there are two cells. The good thing is because the extra part does not have some features of nuclei, the prediction score is lower than the true nuclei, thus some of these false predictions can be avoided by setting a proper threshold.

**Figure 8:**
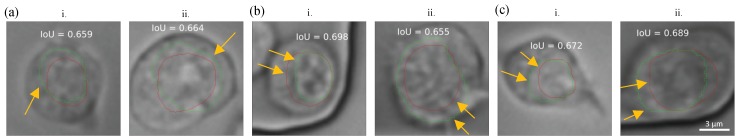
Representative examples of errors in the mask due to (**a**) ambiguous boundaries, (**b**) mismatched size or (**c**) shifted boundaries. Green lines and red lines denote masks from experts and Mask RCNN, respectively.

Figure 8 shows the bad samples with low IoU after the nuclei is correctly detected. There are three possible reasons of the mask error:

1. There is no clear boundary from the phase contrast image, illustrated by the arrows.
2. Predicted masks and ground truth masks are located at the same place, but their size is different. For (b). i, the nucleus is smaller than predicted but for (b). ii, the nucleus is larger and almost overlaps with the cell boundary.
3. Both boundaries from Mask RCNN and experts lie on the area where intensity changes, but they are not matched with each other. The predicted boundary may come from other organelles, thus the center may not be aligned such as (c). ii

Unlike nuclei segmentation from nuclei channel, nuclei do not have obvious boundaries. The network has to learn other features such as nuclei area are kind of “dirty”. This is a challenging problem, improve the image quality or the label accuracy can help.

## 4. Discussion

In this report, we proposed and validated our hypothesis that phase contrast microscopy images contain sufficient information to identify and track nuclei within lymphocytes, with the aid of machine learning. We accomplished this task by training our network against fluorescent labeled nuclei obtained by staining with the DAPI dye. We cross-validated our results by comparisons against a ground-truth, manually validated expert data set, and achieved the IoU of **0.735**. For comparison, the expert validated data and the blob detection method used for detecting the fluorescent nuclei achieved an IoU of 0.842. The relatively high degree of overlap between the phase image and the expert labeled nuclei suggests that phase image has the requisite information to be able to predict the location of the DAPI staining with high accuracy and sensitivity. Our method thus enables the detection of nuclei without the need for exogenous labeling or for the need for phototoxic imaging.

To the best of our knowledge, this is the first study in nuclei segmentation for motile cells like lymphocytes. Conventionally, fluorescent labeling has been used to mark and identify subcellular organelles. Using the nucleus as the example, the traditional method to segment nucleus is Otsu’s thresholding method [12] followed by seeded watershed [19,20]. With the development of the image processing techniques in the computer vision field, more advanced segmentation method such as U-net was introduced in the field to do semantic segmentation [21]. Semantic segmentation only splits objects from different classes and do not differentiate objects in the same class. By contrast, instance segmentation will give each object under the same category a unique label. Juan [22] extended the Unet model to achieve instance segmentation by adding a new boundary class and applying a connected component labeling algorithm. However, the supervised machine learning method requires a large amount of annotated samples which is usually time consuming. Moreover, in time-lapse imaging experiments, the fluorescent signals fade as a function of time.

Our biggest contribution is to detect the nuclei from the phase contrast image instead of stained channels. Although the problem is much more difficult as the edge of nuclei in the phase contrast images are not very clear, with the power of the deep learning method, we can do this very well. We implemented a two-stage workflow during the training: generation of instance masks from stained images and then using these to train a segmentation model based on phase contrast images. Thus there are two types of errors when we compare the masks from Mask RCNN and experts from the training perspective: (a) mask label error (Between blob detection and expert) The IoU difference is 1 − 0.842 = 0.158, which means we still have some space to improve the accuracy of mask label. (b) instance segmentation error (Between Mask RCNN and blob detection). The first mask label error is minimized by using a small ground truth validation set to choose the best parameters for blob detection method. The second segmentation error is taken care of by the Mask RCNN algorithm. Since the training data and test data are distinct, the result shows the ability of Mask RCNN on finding the pattern from phase contrast image to ground truth nuclei masks on time-lapse data.

As we can see, there are still some errors such as false positive detection or inaccurate segmentation. Perhaps the easiest solution is to improve the image quality because some images are not in optimal focus. And high-resolution images can provide us more details about the boundary of those nuclei. One possible way is to use quantitative phase contrast imaging instead of Nomarski differential phase contrast imaging. Several numerical techniques are developed to achieve the digital holography which provides us more useful information such as digital holographic features [23,24]. Quantitative phase imaging can be used in understanding the pathophysiology of diseases [25] and resolving neuronal network activity and spine dynamics [26]. Many machine learning techniques have been proved working well with quantitative phase images, such as logistic regression or k-nearest neighbor classification method in detecting and staging the red blood cells [27] and detecting macrophage activation at single-cell level [28]. But those traditional machine learning methods always require hand-design features which is not the case for deep learning. The combination of deep learning with quantitative phase imaging methods is a logical extension of our work.

Another possibility to improve the detection and segmentation accuracy is to leverage the temporal information within time lapse videos. Adjacent images in a time series often have strong correlations. They can serve as an additional source of correlation to resolve ambiguities caused by overlapping cells of abnormal cell shapes. Our recent work [29] supports this hypothesis by showing that the temporal information in time lapse videos can significantly improve the cell apoptosis classification accuracy. Finally, our pipeline can be extended to detect and segment other subcellular organelles like mitochondria. We expect that adding more subcellular organelles will also improve the accuracy of nuclei results. This is because each task can benefit from the features learned for other tasks. In other words, the cross-benefit is unavailable when a task is performed independently of the others. This effect is well-known in multitasks learning research.

Immunotherapy infusing genetically modified T cells have altered the landscape of cancer treatment with the promise of complete and durable responses in leukemia [30,31]. The manufacture of genetically modified cells as drugs has given birth to a completely new set of challenges [32]. It is essential to be able to profile the functionality of the genetically modified cells prior to infusion and this, in turn, implies the ability to study the many different aspects of dynamic T-cell function including cytokine secretion and cytotoxicity [33,34]. One of the major challenges to studying cellular function in a dynamic manner using timelapse imaging is the ability to have robust algorithms that are able to segment and track the cells accurately [35]. Our paper demonstrates a critical advance in this domain by enabling the detection of not just cellular boundaries but also nuclear boundaries within individual cells. We have recently reported the ability to detect apoptosis within individual cells using just the phase image [29] and thus we are developing the toolkit of algorithms essential to be able to advance cellular immunotherapy. Broadly, we envision the experimental methods and the associated advances in the detection of subcellular organelles will enable the identification of T cells associated with clinical benefit in the context of adoptive cellular immunotherapy.

## Figures and Tables

**Table 1:** Mask generation accuracy with traditional blob detection method. The IoU was computed against a small ground truth mask set. E0, E4, E6 represent the number of erosion pixels are 0, 4 and 6 respectively. E4 masks are used later for training Mask RCNN.

		Otsu	E0	E4	E6
**Mask generation**	IoU	0.653	0.538	**0.842**	0.738
	Instance segmentation	No	Yes	Yes	Yes

**Table 2:** The mean Average precision is given for different IoU thresholds based on detected bounding boxes or detected masks.

	Based on	AP_30	AP_40	AP_50	AP_60	AP_70
**Mask RCNN**	Bounding boxes	0.930	0.906	0.821	0.668	0.365
**Mask RCNN**	Masks	0.922	0.893	0.793	0.568	0.247

## Abbreviations

The following abbreviations are used in this manuscript:

Mask RCNN	Mask Reginal Convolutional Neural Network
IoU	Intersection Over Union
AP	Average Precision
FPN	Feature Pyramid Network
RPN	Region Proposal Network
FG	Foreground
BG	Background
FC	Fully-connected
TP	True Positive
FN	False Negative
TIMING	Timelapse Imaging Microscopy in Nanowell Grids
